# Over-Prescription of Antibiotics for Pulpitis: A Systematic Review and Meta-Analysis of Cross-Sectional Surveys

**DOI:** 10.3390/antibiotics15010013

**Published:** 2025-12-20

**Authors:** Vanessa Delgado-Giugni, María León-López, Isabel Crespo-Gallardo, Juan J. Saúco-Márquez, Paloma Montero-Miralles, Jenifer Martín-González, Daniel Cabanillas-Balsera, Juan J. Segura-Egea

**Affiliations:** Department of Stomatology (Endodontics Section), School of Dentistry, University of Sevilla, C/Avicena s/n, 41009 Sevilla, Spain; vanessadg_141@hotmail.com (V.D.-G.); mleon12@us.es (M.L.-L.); icrespo@us.es (I.C.-G.); jjsauco@us.es (J.J.S.-M.); palmonmir@us.es (P.M.-M.); jmartin30@us.es (J.M.-G.)

**Keywords:** antibiotics, apical abscess, apical disease, apical periodontitis, endodontic infection, meta-analysis, periapical disease, prescription, systematic review

## Abstract

**Background:** Pulpitis requires operative dental treatment, and antibiotics are not indicated. Nevertheless, inappropriate antibiotic prescribing persists worldwide. This systematic review and meta-analysis evaluated the prevalence of antibiotic prescription for pulpitis among dentists. **Methods:** A systematic search of MEDLINE/PubMed, Web of Science, Scopus, Embase, and ProQuest (2015–2025) was performed according to PRISMA guidelines. Observational studies reporting the proportion of dentists prescribing systemic antibiotics for pulpitis were included. Random-effects meta-analyses estimated pooled prevalence for all clinicians, general dental practitioners (GDPs), and endodontists (ENs). Risk of bias was assessed using a modified Newcastle–Ottawa Scale, and certainty of evidence was rated with GRADE. **Results:** Twelve cross-sectional studies met the inclusion criteria, including 3189 dentists. The overall pooled prevalence of antibiotic prescribing for pulpitis was 19.2% (95% CI: 10.4–32.6%), with very high heterogeneity (I^2^ = 98%). GDPs exhibited significantly higher prescribing rates (26.9%, 95% CI: 14.9–43.5%; I^2^ = 98%) compared with ENs (5.1%, 95% CI: 1.2–19.2%; I^2^ = 92%). Sensitivity analysis excluding two high-prevalence studies reduced the pooled estimate to 13.3% (95% CI: 8.0–21.3%) but heterogeneity remained substantial (I^2^ = 95%). Most studies showed moderate-to-high risk of bias, and the certainty of evidence was graded as very low due to inconsistency, indirectness, imprecision, and potential publication bias. **Conclusions:** Approximately one in five dentists prescribe antibiotics for pulpitis, despite strong guideline recommendations against their use. However, certainty of evidence was very low. Marked variability across regions and clinical profiles highlights persistent gaps in diagnostic accuracy, access to emergency dental care, and antibiotic stewardship. Targeted education, improved urgent care pathways, and strengthened antimicrobial stewardship programs are needed to reduce unnecessary antibiotic use in pulpitis.

## 1. Introduction

Optimizing the prescription and use of antibiotics in dentistry has become a public health priority as antimicrobial resistance increases. In endodontics, pulpitis is a common cause of emergency acute dental pain [1], with conservative local dental treatment (vital pulp therapy or root canal treatment) being the therapeutic option in most cases.

Pulpitis is, by definition, a localized inflammatory condition of the vital dental pulp and does not cause fever, disseminated infection, or systemic involvement [1]. The presence of fever, malaise, lymphadenopathy, or other systemic signs indicates a change in diagnosis, as these features are not compatible with pulpitis but rather with pulpal necrosis complicated by an acute apical abscess.

In cases of acute apical abscess associated with systemic involvement (e.g., fever, lymphadenopathy, general malaise) or when the infection is spreading toward cellulitis, the use of systemic antibiotics is clinically indicated. In accordance with the clinical guidelines of both the American Dental Association (ADA) [2] and the European Society of Endodontology (ESE) [3], antibiotics should be prescribed as an adjunct to local operative management of the abscess (e.g., drainage and/or endodontic treatment), and maintained until systemic signs and symptoms have resolved. Antibiotics should never be used as a substitute for definitive local treatment.

However, numerous surveys conducted in different countries show that many dentists continue to prescribe antibiotics for the treatment of pulpitis [4,5], despite little or no improvement in pain outcomes compared to placebo, when surgical care and appropriate analgesics are provided [6].

This persistent gap between guidance and practice has been documented across regions and practice settings [7]. Observational studies and surveys indicate substantial rates of inappropriate dental antibiotic use overall and identify irreversible pulpitis as a recurring scenario for overprescribing [8]. Population-level analyses further suggest that dentistry’s contribution to community antibiotic consumption has grown over recent decades, underscoring the potential impact of targeted stewardship interventions in dental care [9,10]. Qualitative and mixed-methods research points to multifactorial drivers—including diagnostic uncertainty, limited access to urgent operative care, perceived patient expectations, and risk-averse habits—that can influence clinicians toward prescribing even when evidence is unfavorable [11].

At the same time, recent cross-sectional work reveals variable awareness of stewardship principles among dentists, yet also a willingness to modify practice if clear local policies and education are implemented [4,12]. Studies focused on endodontic infections demonstrate heterogeneity in drug choice, duration, and indications, reflecting uncertainty about when antibiotics are appropriate adjuncts to dental conservative treatment and when they are not [13]. Collectively, these findings suggest that aligning real-world prescribing with best evidence could meaningfully reduce unnecessary antibiotic exposure without compromising patient outcomes in pulpitis-related pain.

Given the clinical frequency of pulpitis presentations [1] and the availability of high-quality guidance discouraging routine antibiotics, there is a compelling need to synthesize contemporary data on dentists’ prescribing habits specifically in pulpitis scenarios. This study aims to carry out a systematic review with meta-analysis to investigate the global pattern of antibiotics prescription by dentists in the treatment of pulpitis.

## 2. Materials and Methods

This systematic review is reported using the Preferred Reporting Items for Systematic Reviews and Meta-Analyses (PRISMA 2020) guidelines [14]. A protocol was registered at international prospective register of systematic reviews (PROSPERO) with code CRD42023431788. The registered protocol was originally developed to assess antibiotic prescribing in apical periodontitis. For the present review, the target condition was refined to pulpitis, and the inclusion and exclusion criteria were accordingly adapted to reflect this diagnostic focus. All other methodological aspects of the protocol—including the search strategy, study design eligibility, data extraction procedures, risk-of-bias assessment, and statistical analysis plan—were applied as originally registered.

This study was also conducted following the methodological guidance for systematic reviews of observational epidemiological studies reporting prevalence and cumulative incidence data [15].

### 2.1. Review Question

The review question was structured according to the CoCoPop (Condition, Context, and Population) framework [15], as follows: *what is the prevalence of antibiotic prescribing (Condition) among dentists (Population) in cases of pulpitis (Context)?*

The main outcome was the proportion of dentists prescribing antibiotics in the management of pulpitis. Pulpitis was defined as “A clinical diagnosis based on subjective and objective findings indicating that the vital inflamed pulp is incapable of healing”, according to the AAE Glossary of Endodontic Terms [16] and previous studies [17,18,19,20].

### 2.2. Eligibility Criteria

The inclusion and exclusion criteria were defined according to the CoCoPop (Condition, Context, and Population) framework [15], which is specifically designed for structuring systematic reviews of prevalence data.

#### 2.2.1. Inclusion Criteria

Condition (Co): studies assessing the prescription of antibiotics by dentists for the management of pulpitis. Eligible studies were required to report the prevalence or proportion of antibiotic prescribing.

Context (Co): dental clinical settings, including public and private dental practices, general dentistry, endodontic specialty clinics, and emergency dental services. No restrictions were applied regarding geographic region or healthcare system.

Population (Pop): licensed dentists involved in clinical practice, regardless of specialty, sex, age, or years of experience. Studies including both general practitioners and specialists (e.g., endodontists) were considered eligible if they reported antibiotic prescribing behavior for each one.

Study design: observational studies (cross-sectional surveys, descriptive or analytical studies) reporting quantitative data on the frequency or percentage of antibiotic prescribing in cases of pulpitis were included.

#### 2.2.2. Exclusion Criteria

The following studies were excluded: surveys conducted exclusively among undergraduate or preclinical dental students; experimental or interventional studies (e.g., clinical trials); systematic reviews without original prevalence data; studies focusing exclusively on other dental infections (e.g., apical periodontitis, apical abscesses, pericoronitis, alveolitis) without separate data for pulpitis; and studies evaluating antibiotic prescribing for surgical procedures or postoperative prophylaxis without specific data on pulpitis cases. Reports lacking full-text access or sufficient information to calculate the prevalence of antibiotic prescribing were excluded, as well as animal studies, laboratory research, or simulation-based studies without real clinical data.

### 2.3. Search Strategy and Information Sources

The investigators independently performed electronic searches in PubMed/MEDLINE, Web of Science, Scopus, Embase and Proquest databases (Table 1). The literature search was restricted to studies published from 2015 onward. This time frame was selected to capture contemporary antibiotic prescribing practices in dentistry, following the publication of the World Health Organization Global Action Plan on Antimicrobial Resistance in 2015 [21], which marked a global milestone in the implementation of antimicrobial stewardship policies. Studies published prior to this date may reflect outdated educational frameworks, regulatory environments, and prescribing behaviors that are less representative of current clinical practice. Consequently, studies published prior to 2015 were considered less relevant to current prescribing patterns and stewardship standards. As the primary aim of this review was to estimate the current prevalence of antibiotic prescribing for pulpitis, rather than to assess historical trends, this restriction was considered methodologically appropriate. Moreover, this time frame ensured that the included studies reflected contemporary prescribing behaviors consistent with current principles of antibiotic stewardship in dental practice.

The search was conducted without language restrictions and was updated until November 2025. The search strategy combined controlled vocabulary (MeSH or Emtree terms, depending on the database) and free-text keywords related to pulpitis, antibiotic use, and dental prescribing practices. The same search string was used for all databases, with minor adjustments to syntax and field codes to comply with the specific requirements of each database interface (Table 1). The literature search in all databases was completed on 5 November 2025.

Three authors (J.J.S-E., V.D.-G., and M.L.-L.) independently selected articles. Duplicates were removed using automated detection in the reference manager Mendeley and manual verification. The reference lists of the included articles were also manually searched to identify additional relevant studies. All three reviewers examined the titles and abstracts of the retrieved records to assess their eligibility, followed by full-text evaluation of the potentially relevant articles to decide on their inclusion or exclusion. Disagreements regarding study eligibility were resolved through discussion and consensus. Full texts of all selected studies were subsequently obtained. In addition, manual searches were performed in selected journals and reference lists of relevant publications to ensure comprehensive coverage.

### 2.4. Data Extraction

Two of the authors (J.M.-G. and V.D.-G.) were responsible for data extraction, while three reviewers (D.C-B., J.J.S.-M., and J.J.S-E) verified the tabulated data to ensure the absence of typo errors and carried out the analysis of the articles; the articles in disagreement were discussed. The following details were extracted from the studies: author and year of publication, country, percentage of respondents, prescriber (general practitioner or endodontist), and percentage of antibiotic prescription in cases of pulpitis.

### 2.5. Data Synthesis and Analysis

The primary outcome variable analyzed was the percentage of antibiotic prescribers among dentists. To determine the pattern of antibiotic prescription a meta-analysis was performed using OpenMeta [Analyst] software, version 10.10 [22], applying the DerSimonian–Laird random-effects model for binary data. Proportions were logit-transformed to stabilize variance and better approximate the normality assumptions required for the statistical models. Forest plots were generated to provide a graphical representation of the overall percentage of antibiotic prescriptions. Subgroup analysis was performed for general dental practitioner (GDP) and specialist in Endodontics (EN).

Heterogeneity across studies was assessed using Higgins I^2^ statistic [23], with values between 25 and 50% interpreted as low, 50–75% as moderate, and greater than 75% as indicating high heterogeneity. A *p*-value of 0.05 was considered statistically significant.

To assess the robustness of the pooled estimate, a sensitivity analysis was conducted by excluding studies with markedly elevated prescribing rates.

Publication bias was assessed through visual inspection of a funnel plot and formally tested using Egger’s regression method, which evaluates asymmetry by regressing the standard normal deviate on study precision. A *p*-value < 0.05 for the intercept was considered indicative of significant small-study effects or potential publication bias.

### 2.6. Risk of Bias Assessment

Each included study was independently assessed for methodological quality by three reviewers (M.L-L., D.C.-B., and V.D.-G.) using a modified version of the Newcastle–Ottawa Scale (NOS) [24] adapted for prevalence studies [25,26] (Table 2). This modified scale better fits the review’s focus on survey-based prevalence studies evaluating dentists’ prescribing behavior. In cases of disagreement, consensus was reached through discussion.

The adapted tool comprised two domains (1) sample selection and representativeness, and (2) measurement and data analysis. Each item meeting the criterion was awarded one point, with a maximum of 8 points indicating the lowest risk of bias. The overall risk of bias was determined according to the total NOS score: scores of 0–4 indicated high risk of bias, 5–6 indicated moderate risk, and 7–8 indicated low risk of bias.

### 2.7. Certainty of Evidence (GRADE Assessment)

The Grading of Recommendations, Assessment, Development and Evaluation (GRADE) approach [27] was applied to assess the overall certainty of the evidence. This method establishes an initial level of certainty based on the study design and subsequently evaluates several domains, including risk of bias, inconsistency, indirectness, imprecision, publication bias, dose–response relationship, confounding factors, and magnitude of effect, to determine the final certainty rating. Under this framework, moderate or high certainty indicates a medium or high level of confidence in the estimated effect, respectively. In contrast, low or very low certainty reflects limited or minimal confidence in the reliability of the findings [28].

## 3. Results

**Figure 1:**
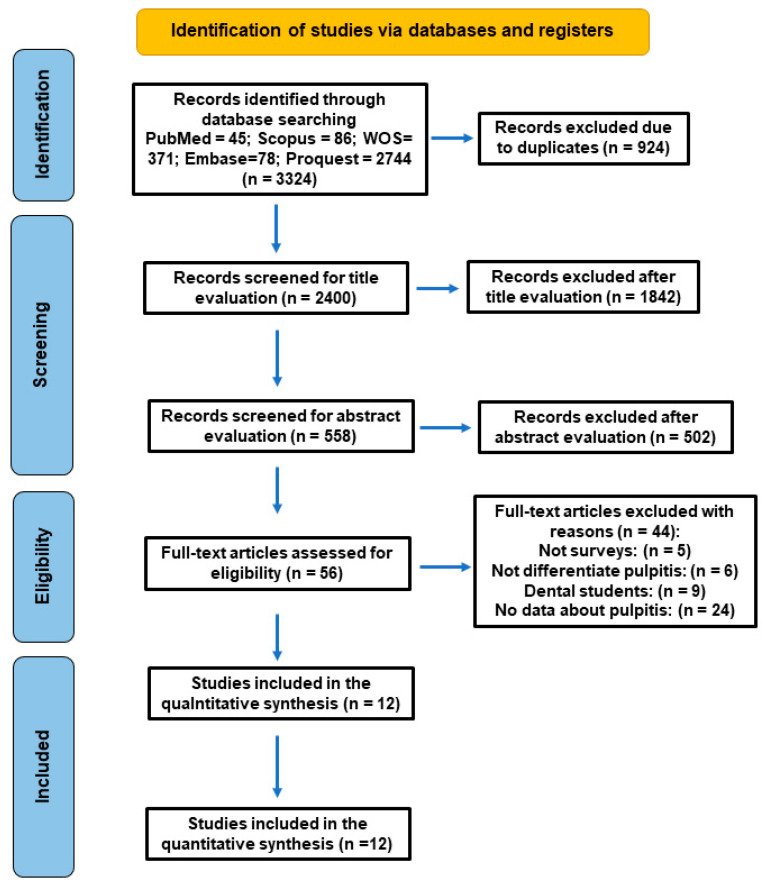
Flowchart of the search strategy following the PRISMA 2020 guidelines for systematic reviews and meta-analyses.

The baseline search recovered 3329 titles (Figure 1). After the removal of duplicates (*n* = 924), 2400 articles remained, of which 1842 were excluded after title evaluation, and another 502 were also excluded after reading abstract for being unrelated to the topic, and 56 were selected for full text. Then, 44 studies were excluded due to predefined eligibility criteria (Table 3).

Nine studies were removed because they were conducted on dental students rather than licensed dentists, and therefore did not reflect the prescribing behavior of practicing clinicians. Five studies were excluded because they did not report survey-based data, instead relying on clinical records or hospital databases, which did not meet the methodological requirements of this review. Six additional studies failed to specify pulpitis as a distinct diagnostic category or did not differentiate it from other endodontic conditions, preventing extraction of condition-specific prescribing outcomes. The largest group (*n* = 24) excluded studies that, despite meeting other clinical or methodological criteria, did not report the percentage of antibiotic prescriptions for cases of pulpitis, making them incompatible with the primary outcome of interest.

These exclusions ensured that only studies providing clear diagnostic definitions and explicit antibiotic-prescription data for pulpitis were included in the qualitative synthesis and subsequent analyses.

Finally, twelve studies fulfilled the inclusion criteria and were selected for systematic review and meta-analysis [4,29,30,31,32,33,34,35,36,37,38,39].

### 3.1. Characteristics of the Included Studies

The characteristics of the 12 cross-sectional surveys included in the systematic review are summarized in Table 4.

All selected studies were published between 2015 and 2024 and collectively represent a wide geographical distribution, encompassing countries from Asia (Saudi Arabia, Pakistan, Kuwait, Turkey), Europe (Spain, Serbia), and South America (Brazil, Colombia). The studies surveyed primarily general dental practitioners, with a smaller number involving endodontists [31,32,34], providing a broad overview of prescribing patterns across different professional profiles and healthcare settings.

Sample sizes and response rates varied markedly across studies. Overall, total samples ranged widely—from 73 to 13,853—highlighting substantial heterogeneity across studies. Five of the studies showed high participation rates, above 90% [4,29,32,33,39]. Four others showed moderate rates (53–79%) [30,31,36,37]. In contrast, three studies showed low engagement [34,35,38].

Regarding the diagnosis of pulpitis, most authors provided explicit clinical diagnostic criteria for pulpitis, generally based on signs and symptoms such as pain characteristics, duration, and response to stimuli, reporting clear diagnostic descriptions of pulpitis [4,29,32,33,34,37]. Three studies reported detailed diagnostic frameworks, often presented through clinical scenarios and aligned with international guidelines (ESE/AAE) for both endodontists [31], as well as for GDPs [30,31,36]. In contrast, two studies [35,38] did not report any specific diagnostic criteria, referring to pulpitis merely as a diagnostic label without supporting clinical detail. Overall, while the majority of studies offered sufficient diagnostic information, the lack of standardized criteria in a subset of publications may limit comparability across samples.

### 3.2. Pattern of Antibiotic Prescription in Pulpitis

The pattern of antibiotic prescription in pulpitis is shown in Table 5. Across the included studies, amoxicillin—alone or combined with clavulanic acid—was consistently the most frequently prescribed first-line antibiotic for pulpitis. Most general dental practitioners selected plain amoxicillin as their first choice [4,29,31,33,37,38,39], while some studies reported amoxicillin-clavulanic acid as the preferred initial option [30,32,36]. Among endodontists, amoxicillin also predominated as the first-line agent [31,32,34]. Regarding second-line therapies, the patterns varied slightly but commonly included amoxicillin-clavulanic acid, metronidazole, or azithromycin. Clindamycin was systematically identified as the antibiotic of choice for patients with penicillin allergy.

Concerning the duration of the antibiotics treatment, most clinicians prescribed antibiotics for a duration of 5 to 7 days, with 7 days emerging as the most frequent regimen. This pattern was consistently observed among general dental practitioners and endodontists in Colombia [31], where over three-quarters of respondents selected a 7-day course. Similar trends appeared in studies from Pakistan [33] and Saudi Arabia [29,38], in which a 5–7-day prescription was the predominant choice for pulpitis-related conditions. Shorter courses of 3 days were reported only infrequently, and extended regimens of 10 days or more were uncommon, representing a small minority of respondents across the included studies. Overall, the duration of therapy showed low variability between countries and prescriber types, reflecting a widespread preference for standard 5–7-day antibiotic courses in endodontic infections.

Antibiotic prescribing in cases of pulpitis showed substantial variability across the included studies, reflecting marked differences in clinical decision-making among dentists worldwide. Very low prescribing rates (1.1–1.3%) were reported by endodontists in Brazil [34] and by general practitioners in Serbia [35], respectively.

Similarly, several studies showed low-to-moderate levels (9.2–28.0%) [31,32,38,39]. Three studies reported intermediate prescribing levels (27.3–44.0%) [4,36,37]. In contrast, markedly higher rates were observed in Pakistan where more than 50% of dentists prescribed antibiotics in cases of pulpitis [30,33]. These findings reveal a wide spectrum of clinical attitudes, from strict adherence to evidence-based guidance discouraging antibiotics in pulpitis, to substantial overprescribing in certain regions, suggesting persistent misconceptions regarding the role of antibiotics in managing pulpal inflammation.

### 3.3. Meta-Analysis of Antibiotic Prescription for Pulpitis by Dentists

**Figure 2:**
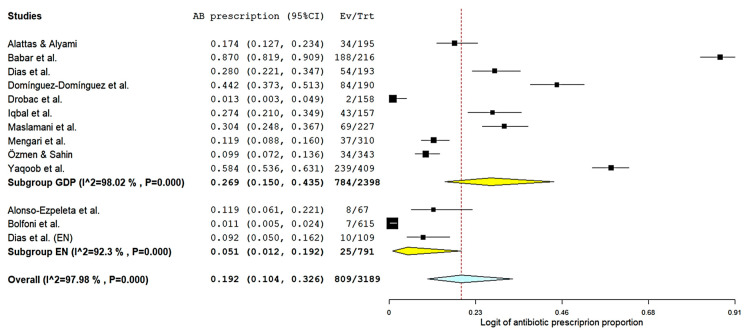
Forest plot of the meta-analysis on the proportion of antibiotic prescriptions in cases of pulpitis. Individual study estimates with 95% confidence intervals are shown, grouped by subgroups (GDP: general dental practitioners, yellow; EN: endodontists, yellow). Pooled estimates were calculated using random-effects models. The red dashed line represents the overall logit-transformed antibiotic prescription proportion (blue diamond) [4,29,30,31,32,33,34,35,36,37,38,39].

A random-effects meta-analysis was conducted using the 12 studies that reported the proportion of clinicians prescribing antibiotics for cases of pulpitis (Figure 2, overall). For each study, the percentage of prescribers was calculated using the number of respondents extracted from Table 4. The results of the Dias et al. (2022) [31] study are shown separately for GDPs and ENs. After logit transformation of proportions and estimation of variances using the binomial approximation, a DerSimonian–Laird random-effects model was applied to account for substantial variability across settings.

The pooled estimate showed that 19.2% of clinicians prescribed antibiotics for pulpitis (95% CI: 10.4–32.6%; *p* < 0.001), indicating that approximately one in five practitioners reported using antibiotics in this non-indicated scenario.

Assessment of heterogeneity demonstrated that variability across studies was considerable. The Q statistic was highly significant (Q = 580.7; df = 12; *p* < 0.0001), confirming that the dispersion of effect sizes was greater than expected by chance. The between-study variance was large (τ^2^ = 1.59, logit scale), and I^2^ reached 98%, indicating that nearly all observed variability reflected true differences in prescribing behaviors rather than sampling error. This very high heterogeneity is consistent with the wide range of reported prescribing rates, which spanned from near-zero values in some European and South American samples to markedly elevated rates (above 50–80%) in several South Asian and Middle Eastern cohorts.

### 3.4. Meta-Analysis of Antibiotic Prescription for Pulpitis by Dental General Practitioners

A separate random-effects meta-analysis was conducted including only the studies in which prescribers were general dental practitioners (GDPs) (Figure 2, subgroup GDP). Ten studies contributed data to this analysis [4,29,30,31,33,35,36,37,38,39]. Logit-transformed proportions and binomial variances were used, and a DerSimonian–Laird random-effects model was applied. The pooled estimate indicated that 26.9% of GDPs prescribed antibiotics for pulpitis (95% CI: 14.9–43.5%), representing roughly one in four general practitioners.

Assessment of heterogeneity showed pronounced variability across studies. The Q statistic was highly significant (Q = 454.3; df = 9; *p* < 0.0001), demonstrating that the dispersion of effect sizes was substantially greater than expected by chance alone. The between-study variance was considerable (τ^2^ = 1.35, logit scale), and the corresponding I^2^ was 98%, indicating extreme heterogeneity. This high level of inconsistency reflects the wide differences in antibiotic prescribing behavior among GDPs worldwide.

### 3.5. Meta-Analysis of Antibiotic Prescription for Pulpitis by Endodontists

A separate random-effects meta-analysis was performed including only the studies in which the prescribers were endodontists [31,32,34] (Figure 2, subgroup EN). The pooled estimate showed that 5.1% of endodontists prescribed antibiotics for pulpitis (95% CI: 1.2–19.2%; *p* < 0.001), indicating that antibiotic use for this condition was generally uncommon among specialists.

Statistical heterogeneity, however, was substantial. The Q test was highly significant (Q = 26.1; df = 2; *p* < 0.0001), and the between-study variance was large (τ^2^ = 1.60 on the logit scale). The corresponding I^2^ was 92%, indicating that most of the observed variability reflected genuine differences in prescribing behavior across endodontist samples rather than random error.

### 3.6. Sensitivity Analysis and Publication Bias

A sensitivity analysis was conducted to evaluate the robustness of the pooled estimate by excluding the two studies with markedly elevated prescribing rates [30,33]. After their removal, the pooled proportion of clinicians prescribing antibiotics for pulpitis decreased substantially from 19.2% in the main analysis to 13.3% (95% CI: 8.0–21.3%). Despite the change in magnitude, substantial heterogeneity persisted (Q = 213.3; df = 10; *p* < 0.0001; τ^2^ = 0.83; I^2^ = 95%), indicating that true variability across study populations remained pronounced even after excluding outliers. This suggests that while extreme high-prescribing cohorts exerted notable influence on the overall prevalence, considerable structural differences across settings continued to drive variability in antibiotic prescribing behaviors.

**Figure 3:**
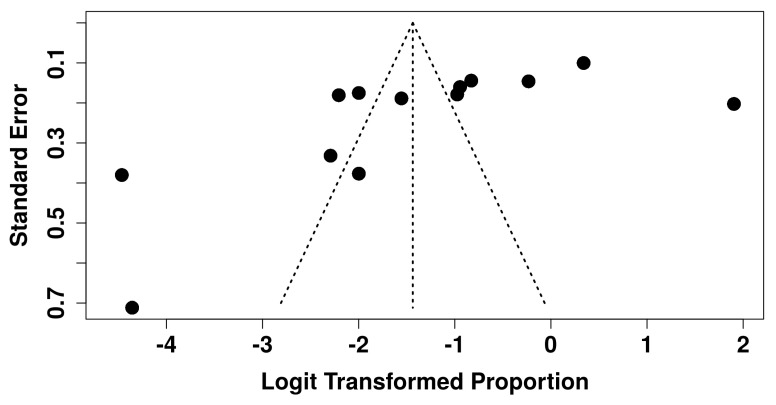
Funnel plot of the included studies, plotting the logit transformed proportion of antibiotic prescribing against its standard error. The vertical line represents the pooled random-effects estimate. Asymmetry may indicate small-study effects or publication bias.

Publication bias and small-study effects were examined to assess whether reporting asymmetries might have influenced the pooled estimates. A funnel plot (Figure 3) revealed some degree of asymmetry, with smaller studies tending to deviate from the central distribution pattern. This visual impression was supported by Egger’s regression test, which showed a statistically significant intercept (β_0_ = −10.13, SE = 4.18, t = −2.42, *p* = 0.034), indicating potential small-study effects or selective reporting. The Doi plot produced an LFK index of +1.55, corresponding to minor asymmetry. Although both indicators suggest the presence of publication bias, these findings should be interpreted cautiously given the extremely high heterogeneity observed across the included studies, which can produce apparent asymmetry even in the absence of true reporting bias. Taken together, the sensitivity and publication bias analyses indicate that while the direction of the findings is robust, the magnitude of the pooled prevalence should be interpreted in light of persistent heterogeneity and potential small-study effects.

### 3.7. Risk of Bias Assessment

The risk of bias assessment, conducted using the modified Newcastle–Ottawa Scale for cross-sectional studies, revealed substantial variability in methodological quality among the included studies (Table 6). Overall scores ranged from 2 to 8 out of 8, corresponding to risk-of-bias levels from high to low. A considerable number of studies were judged to have high risk of bias [30,31,33,35,36,38], mainly due to concerns related to sampling representativeness, lack of sampling frame clarity, or unclear measurement validity.

A second group demonstrated moderate risk of bias [4,29,32,34,37], typically reflecting partial fulfillment of representativeness and outcome-assessment criteria. Only one study [39] reached a low risk of bias, supported by clear sampling procedures and consistent reporting across NOS domains. Overall, the body of evidence presents a predominantly moderate-to-high risk of bias, indicating that methodological limitations should be carefully considered when interpreting the pooled estimates.

### 3.8. GRADE Assessment of Evidence Quality

The certainty of the evidence regarding the prevalence of antibiotic prescription for pulpitis was assessed using the GRADE framework (Table 7). Overall, the body of evidence was judged to be of very low certainty. Most included studies were cross-sectional surveys (very low certainty) with important methodological limitations, and the majority were rated as having moderate to high risk of bias, with only one study meeting criteria for low risk. This resulted in downgrading for risk of bias.

There was very serious inconsistency, with effect estimates ranging from near-zero prescribing in several European and South American samples to more than 80% in certain South Asian and Middle Eastern cohorts. This variability was reflected in extreme statistical heterogeneity (I^2^ = 98%, *p* < 0.0001), and the lack of identifiable sources of heterogeneity warranted further downgrading.

The evidence was also downgraded for indirectness, as all studies relied on self-reported prescribing practices rather than observed clinical behavior. Some studies did not clearly distinguish pulpitis from other diagnoses when reporting antibiotic use, and substantial variability in clinical training, health system structure, and prescribing regulations across countries further reduced direct applicability.

Despite a large total sample across studies, several individual samples were small or reported few events, and the pooled estimate showed a wide confidence interval (10.4–32.6%), leading to downgrading for imprecision.

Finally, publication bias was considered likely. The funnel plot showed some asymmetry, and Egger’s regression test was statistically significant (*p* = 0.034). The Doi plot yielded an LFK index of +1.55, indicating minor asymmetry, although the very high heterogeneity complicates interpretation. These findings justified an additional downgrade for publication bias.

Taken together, the cumulative downgrades across all domains indicate that the certainty of the evidence is very low, meaning that the true prevalence of antibiotic prescribing for pulpitis may differ substantially from the pooled estimate obtained in this review.

## 4. Discussion

This systematic review and meta-analysis evaluated global patterns of antibiotic prescribing for pulpitis. The pooled prevalence of antibiotic use was 19.2%, meaning that nearly one in five clinicians reported prescribing antibiotics for a condition where systemic antimicrobials offer no therapeutic benefit according to current international guidelines, including those of the ADA [40] and ESE [3]. Similar findings have been reported in previous investigations highlighting persistent antibiotic misuse in endodontic emergencies [41,42,43]. The broad prevalence range observed across studies—from near-zero to above 80%—reflects substantial variability in dentists’ diagnostic accuracy, access to urgent dental care, and confidence in providing definitive operative treatment, as well as contextual differences across health systems, patient expectations, and availability of out-of-hours dental services.

Subgroup analyses reinforced these differences: general dental practitioners (GDPs) demonstrated substantially higher prescribing rates (26.9%) than endodontists (5.1%), consistent with other studies showing that specialists generally adhere more closely to evidence-based prescribing recommendations [13,44]. This discrepancy likely reflects differences in postgraduate training, greater familiarity of endodontists with the management of acute dental pain, and increased availability of resources for immediate operative care in specialist settings. These findings underscore the need for targeted educational interventions, clinical decision-support tools, and routine antibiotic stewardship initiatives aimed specifically at GDPs.

While a sensitivity analysis reduced the pooled prevalence to 13.3% after removing outlier studies [30,33], heterogeneity remained considerable, indicating that inappropriate prescribing is driven by structural and contextual factors rather than isolated study anomalies. Persistent high heterogeneity (as reflected in I^2^ values) suggests true variability in clinical practice patterns influenced by factors such as differences in undergraduate curricula [45,46], differences among GDP and endodontists [13], limited emergency care infrastructure [44], and culturally driven prescribing norms [7]. Addressing these systemic contributors is essential for reducing unnecessary antibiotic use globally.

Indicators of publication bias, including a significant Egger’s test and minor asymmetry in the Doi plot, suggest the potential presence of small-study effects. However, as noted in other meta-analyses involving heterogeneous surveys [13,47], high between-study variability can also produce apparent funnel-plot asymmetry. This is particularly relevant for questionnaire-based studies, where smaller samples often arise from settings with atypically high or low prescribing rates, creating artefactual asymmetry. Therefore, caution is warranted in attributing these findings solely to publication bias.

Applying the GRADE approach, the certainty of evidence was classified as very low, reflecting multiple concerns across domains. Downgrading was primarily driven by the high risk of bias in several studies, large inconsistency between estimates, indirectness due to variations in clinical scenarios across countries, and imprecision related to wide confidence intervals. Despite the very low certainty, the consistent pattern of unnecessary antibiotic prescribing observed across diverse settings highlights a clinically relevant problem that warrants urgent attention.

### 4.1. Consistency of the Findings

Previous international reviews and surveillance studies have consistently documented inappropriate antibiotic use in dental settings [41,43,44]. Pulpitis has repeatedly been identified as one of the principal conditions for which antibiotics are unjustifiably prescribed, often due to misconceptions about their analgesic or anti-inflammatory benefit [42,43]. The low prescribing rates among endodontists in this review mirror those reported in specialist surveys [13], reflecting the impact of advanced training on diagnostic precision and confidence in operative management, as well as greater adherence to guideline-based decision-making and a deeper understanding of the inflammatory pathophysiology underlying pulpitis.

Geographical variation observed in our study aligns with previous findings showing higher antibiotic use in regions with limited access to emergency dental care or weaker stewardship frameworks [29,33,44]. These disparities illustrate how differences in healthcare infrastructure, appointment availability, financial constraints, and patient expectations contribute to divergent prescribing behaviors. Moreover, contextual factors such as variable enforcement of clinical guidelines [10], cultural norms around antibiotic use [10,41], and differences in undergraduate training [46] further amplify regional inconsistencies. Together, these findings reinforce that inappropriate antibiotic prescribing is not an isolated issue but a globally observed pattern shaped by system-level and sociocultural influences [10].

### 4.2. Factors Explaining the Variability

Several contextual factors may explain the wide variability found. Firstly, the diagnostic uncertainty. A well-documented barrier to appropriate prescribing [5], often leading clinicians to err on the side of caution. This uncertainty is frequently compounded by inconsistent use of diagnostic criteria, limited availability of vitality testing devices, and variability in clinicians’ confidence when differentiating reversible from irreversible pulpitis. Another factor is the limited access to urgent operative care; in settings where pulpotomy or pulpectomy are not readily available, antibiotics are sometimes used as a perceived “stop-gap” measure [33,44]. The training differences can also explain the variability found. The contrast between GDPs and endodontists observed here mirrors other findings indicating that additional training reduces reliance on antibiotics [13,47], likely by strengthening decision-making skills, enhancing familiarity with emergency endodontic procedures, and promoting adherence to evidence-based guidelines.

Another factor that may also explain, at least in part, the observed variability are patient expectations. Patients frequently associate antibiotics with analgesia or rapid symptom relief, contributing to pressure on clinicians [41]. This dynamic is intensified in healthcare models where patient satisfaction influences clinical decision-making or where communication barriers impede effective explanation of the non-indicated nature of antibiotics. Health system and cultural factors, such as international disparities in regulation, stewardship, and prescribing autonomy have also been highlighted in several cross-national comparisons [12,44]. Differences in the enforcement of stewardship policies, availability of national prescribing guidelines, and societal norms regarding self-medication further contribute to heterogeneous patterns of antibiotic use. These determinants emphasize that antibiotic misuse for pulpitis is multifactorial and embedded within broader systemic and sociocultural contexts.

### 4.3. Clinical Implications

The findings of this review have important implications for clinical practice. Reinforcement of evidence-based guidelines is urgently needed. Established guidance [48] clearly states that antibiotics do not relieve pulpal pain and should not replace operative treatment. Moreover, the European Society of Endodontology (ESE) position statement clearly indicates that antibiotics should not be prescribed for the treatment of pulpitis [3]. Other previous studies have shown that the problem of over-prescription of antibiotics in endodontics not only occurs due to prescription in cases of pulpitis, but over-prescription also extends to the treatment of apical periodontitis and apical abscess [4,10,49]. These patterns collectively demonstrate that antibiotic misuse in dental practice is pervasive and extends across multiple endodontic diagnoses, underscoring the urgent need for system-wide corrective strategies.

Strengthening undergraduate and continuing education is essential, particularly given the higher prescribing rates among GDPs compared with endodontists [13,32]. Some surveys conducted among students show that they finish their dentistry degree studies without having acquired clear and certain knowledge about the prescription of antibiotics in endodontic infections [50,51,52]. Enhancing curricula to include structured training in endodontic diagnosis, emergency management, and antimicrobial stewardship could help reduce inappropriate prescribing from the outset of clinicians’ careers. Continuing education programs should also prioritize practical decision-making algorithms and case-based learning to address persistent gaps among practicing GDPs.

Improved access to urgent dental care is fundamental. Studies show that inappropriate prescribing often reflects barriers to delivering or accessing timely operative treatment [44]. Expanding clinical capacity, extending emergency service hours, and integrating endodontic triage pathways may help reduce reliance on antibiotics as a substitute for definitive care. Enhancing communication strategies to help clinicians address patient misconceptions regarding antibiotics may reduce the perceived pressure to prescribe [41]. Moreover, the integration of dental antibiotic stewardship programs within broader healthcare systems can help standardize prescribing behavior [12,47]. Embedding audit-and-feedback cycles, electronic prescribing alerts, and interdisciplinary collaboration with medical stewardship teams may provide sustained improvements in antibiotic use within dental settings.

### 4.4. Limitations of the Evidence

The evidence synthesized in this review presents several limitations. All included studies were cross-sectional surveys relying on self-reporting, which is prone to recall and social desirability bias. Additionally, many studies lacked representative sampling, as reflected in their moderate or high risk-of-bias ratings (Table 6). The absence of standardized or validated questionnaires across studies further limits comparability and may contribute to variability in reported prescribing practices.

The high heterogeneity between the studies is another limitation. I^2^ values around 98% indicate large between-study variability that could not be fully explained by subgroup analyses. Such substantial heterogeneity likely reflects real-world differences in clinical practice, healthcare infrastructure, and training, but it also reduces the precision of pooled estimates and complicates their interpretation.

Another possible limitations are indirectness: the variability in how pulpitis was defined and reported reduces the direct applicability of findings. Some surveys combined symptomatic and asymptomatic presentations or failed to distinguish between reversible and irreversible pulpitis, limiting the ability to map prescribing behaviors to specific clinical scenarios. Imprecision was also evident, as confidence intervals widths were substantial, and several studies had small sample sizes or low event counts. This imprecision weakens the robustness of pooled prevalence estimates and highlights the need for larger, methodologically rigorous studies.

Regarding the publication bias, both funnel-plot asymmetry and Egger’s test suggest potential small-study effects. However, caution is warranted in interpreting these findings, as observational survey research often displays artefactual asymmetry due to methodological and contextual variability rather than true selective publication. These limitations collectively justify the very low certainty assigned through GRADE. Despite these constraints, the consistency in the direction of findings across diverse contexts strengthens confidence in the overall conclusion that antibiotics are frequently misused for pulpitis.

Finally, an additional limitation of this review is the restriction of the search to studies published from 2015 onward. Although antimicrobial stewardship concepts and recommendations existed prior to this date, older surveys may reflect historical prescribing patterns that differ substantially from current practice. Consequently, this review does not capture long-term temporal trends in antibiotic prescribing for pulpitis, but rather provides a focused assessment of contemporary practice in the post–Global Action Plan era.

### 4.5. Future Directions

Future research should prioritize well-designed clinical observational studies assessing real-world prescribing, representative national or regional sampling, standardized diagnostic criteria for pulpitis, multilevel analyses evaluating system-level drivers of prescribing, and interventions assessing the impact of education and stewardship programs. Such evidence will be essential to addressing inappropriate antibiotic use. In addition, future studies should incorporate validated prescribing indicators and harmonized survey instruments to facilitate cross-country comparisons and meta-analytic synthesis. Longitudinal research is also needed to evaluate trends over time and determine whether stewardship initiatives translate into sustained reductions in inappropriate prescribing. Implementation science approaches may help identify barriers and facilitators to adopting evidence-based practices, while mixed-methods studies could provide deeper insight into clinician decision-making and patient expectations. Finally, collaborative international research networks could play a key role in developing unified protocols and strengthening global surveillance of antibiotic use in endodontics.

### 4.6. Conclusions

This systematic review demonstrates that antibiotics are often prescribed for pulpitis at rates inconsistent with clinical guidelines, with meaningful variation across clinicians and countries. Although the overall prevalence is moderate, the persistence of inappropriate prescribing contributes to unnecessary antibiotic exposure and increased antimicrobial resistance. These findings reinforce that pulpitis remains one of the most frequently mismanaged dental conditions with respect to antimicrobial use, despite long-standing and internationally recognized recommendations against prescribing. Improving diagnostic training, expanding access to emergency dental procedures, and implementing robust antibiotic stewardship strategies are essential steps toward aligning clinical practice with evidence-based care. Such initiatives should be tailored to address both clinician-level and system-level contributors, acknowledging the multifactorial nature of prescribing behaviors and the influence of cultural and structural factors across healthcare systems. Without such measures, inappropriate antibiotic use for pulpitis is likely to persist, undermining global efforts to preserve antimicrobial effectiveness. Given the increasing urgency of combating antimicrobial resistance, optimizing antibiotic use in dentistry should be considered a priority within broader public health frameworks.

## Figures and Tables

**Table 1:** Exact search strings used for each database.

Database	Exact Search String Used	No. of Articles	Date of Last Search
**PubMed/** **MEDLINE**	(pulpitis[MeSH Terms] OR pulpitis[Title/Abstract] OR “irreversible pulpitis”[Title/Abstract]) AND (antibiotic*[Title/Abstract] OR antimicrobial*[Title/Abstract] OR “anti-bacterial agents”[MeSH Terms]) AND (prescribing[Title/Abstract] OR prescription*[Title/Abstract] OR “inappropriate use”[Title/Abstract] OR “overprescription”[Title/Abstract] OR misuse[Title/Abstract]) AND (dentist*[Title/Abstract] OR “dental practitioner*”[Title/Abstract] OR “general dental practitioner*”[Title/Abstract] OR endodontist*[Title/Abstract])	45	From 2015 to 5 November 2025
**Scopus**	(TITLE-ABS-KEY(pulpitis) OR TITLE-ABS-KEY(“irreversible pulpitis”)) AND (TITLE-ABS-KEY(antibiotic*) OR TITLE-ABS-KEY(antimicrobial*) OR TITLE-ABS-KEY(“anti-bacterial agents”)) AND (TITLE-ABS-KEY(prescribing) OR TITLE-ABS-KEY(prescription*) OR TITLE-ABS-KEY(“inappropriate use”) OR TITLE-ABS-KEY(overprescription) OR TITLE-ABS-KEY(misuse)) AND (TITLE-ABS-KEY(dentist*) OR TITLE-ABS-KEY(“dental practitioner*”) OR TITLE-ABS-KEY(“general dental practitioner*”) OR TITLE-ABS-KEY(endodontist*))	86	From 2015 to 5 November 2025
**Web of Science**	(pulpitis OR dental infection) AND (antibiotic OR antimicrobial) AND (prescribing OR prescription) AND (dentist* OR “dental practitioner*” OR “general dental practitioner*” OR endodontist*)	371	From 2015 to 5 November 2025
**EMBASE**	(‘pulpitis’/exp OR pulpitis:ti,ab,kw OR “irreversible pulpitis”:ti,ab,kw) AND (antibiotic*:ti,ab,kw OR antimicrobial*:ti,ab,kw OR ‘antibacterial agent’/exp OR “anti-bacterial agent*”:ti,ab,kw) AND (prescribing:ti,ab,kw OR prescription*:ti,ab,kw OR “inappropriate use”:ti,ab,kw OR overprescription:ti,ab,kw OR misuse:ti,ab,kw) AND (dentist*:ti,ab,kw OR (dental NEXT/1 practitioner*):ti,ab,kw OR (general NEXT/1 dental NEXT/1 practitioner*):ti,ab,kw OR endodontist*:ti,ab,kw OR ‘dentist’/exp OR ‘endodontist’/exp)	78	From 2015 to 5 November 2025
**ProQuest (Grey Literature)**	(pulpitis OR dental infection) AND (antibiotic OR antimicrobial) AND (prescribing OR prescription) AND (dentist* OR “dental practitioner*” OR “general dental practitioner*” OR endodontist*)	2749	From 2015 to 5 November 2025

**Table 2:** Modified Newcastle–Ottawa Scale (NOS) for prevalence studies.

**Domain 1: Sample Selection and Representativeness**
1. Representativeness of the sample: the sample is truly representative of the target population (e.g., national or regional random sample, or all eligible participants included).
2. Sampling frame and selection method: sampling frame is appropriate (e.g., registry, professional association list) and participants are selected randomly or systematically.
3. Sample size justification: sample size is adequately calculated and justified based on expected prevalence or precision level.
4. Non-response bias: response rate ≥ 70%, or analysis demonstrates no significant difference between respondents and non-respondents.
**Domain 2: Measurement and Data Collection**
5. Case/phenomenon definition: the variable of interest (e.g., antibiotic prescription for a specific condition) is clearly defined and consistent across participants.
6. Measurement tool validity: data are collected using validated or standardized instruments (questionnaire, clinical record, or national survey form).
7. Consistency of data collection: the same data collection methods and definitions are applied to all participants.
8. Statistical analysis: appropriate descriptive and inferential analyses are performed, including 95% confidence intervals for prevalence estimates.

**Table 3:** Excluded studies and their reasons for exclusion.

Reasons	Excluded Studies
Studies that did not provide survey data but instead used clinical records or hospital registries (*n* = 5).	Al Asmar et al., 2020 Carlsen et al., 2021 Di Giuseppe et al., 2021 Lalloo et al., 2017 Tanwir et al., 2015
Studies that did not differentiate pulpitis from other endodontic pathologies (*n* = 6).	Baudet et al 2020 Bidar et al., 2015 Gemmell et al 2020 Hamdan et al., 2024 Joji et al., 2024 Khijmatgar et al., 2024
Studies conducted on dental students rather than licensed dentists (*n* = 9).	Arıcan, 2021 Careddu & Duncan, 2021 Danadneh, 2025 Doshi, 2017 Kumar Giri, 2025 Madarati, 2018 Salvadori, 2019 Schneider-Smith, 2023 Strużycka, 2019
Studies without percentages of antibiotic prescription for pulpitis (*n* = 24).	Abdellatif, 2025 Ahsan, 2020 Alzahrani, 2020 Bjelovucic, 2019 Buttar, 2017 Edwards, 2024 Goff, 2022 Jain, 2015 Kalantzis, 2024 Khalab, 2024 Khalil, 2022 Kusumoto, 2021 López-Marrufo-Medina et al., 2022 Mathur, 2023 Peric et al., 2015 Roberts, 2018 Robles Raya, 2017 Rodríguez-Fernández, 2023 Shemesh, 2022 Simon, 2023 Sovic, 2024 Sturrock, 2018 Vengidesh et al., 2024 Yu et al., 2020

**Table 4:** Characteristics of the included studies.

Authors & Year	Country	Prescriber	Total Sample	Percentage of Respondents	Diagnosis of Pulpitis
Alattas & Alyami 2017 [29]	Saudi Arabia	GDP	200	98.0	Clinical diagnostic criteria through signs and symptoms
Alonso-Ezpeleta et al. 2018 [32]	Spain	EN	73	91.0	Clinical diagnostic criteria through signs and symptoms
Babar et al. 2022 [33]	Pakistan	GDP	216	100	Clinical diagnostic criteria through signs and symptoms
Bolfoni et al. 2018 [34]	Brazil	EN	13,853	4.4	Clinical diagnostic criteria through signs and symptoms
Dias et al. 2022 [31]	Colombia	GDP	363	53.2	Clinical scenarios containing complete criteria for pulpitis consistent with ESE and AAE guidelines
Dias et al. 2022 [31]	Colombia	EN	196	55.6	
Domínguez-Domínguez et al. 2021 [4]	Spain	GDP	200	95.0	Clinical diagnostic criteria through signs and symptoms
Drobac et al. 2021 [35]	Serbia	GDP	628	25.2	Only mentions pulpitis as a diagnostic label without describing symptoms or signs
Iqbal et al. 2015 [36]	Saudi Arabia	GDP	200	78.5	Clinical scenarios with detailed clinical information that allow diagnosis of pulpitis
Maslamani & Sedeqi 2018 [37]	Kuwait	GDP	300	75.6	Clinical diagnostic criteria through signs and symptoms
Mengari et al. 2020 [38]	Saudi Arabia	GDP	1500	22.1	Only mentions pulpitis as a diagnostic label without describing symptoms or signs.
Özmen & Şahin 2024 [39]	Turkey	GDP	360	95.3	Clinical cases with descriptions of symptoms of pulpitis
Yaqoob et al. 2024 [30]	Pakistan	GDP	527	77.6	Clinical scenarios with detailed clinical information that allow diagnosis of pulpitis

GDP: general dental practitioner; EN: endodontist.

**Table 5:** Pattern of antibiotic prescription by dentists in the treatment of pulpitis.

Authors & Year	Prescriptors	Respondents	Antibiotics Prescription in Pulpitis (%)	First-Choice Antibiotics	Second-Choice Antibiotics	Antibiotics in Allergic Patients	Duration (Days)
Alattas & Alyami 2017 [29]	GDP	195	34 (17.5)	Amoxicillin	Amoxicillin + clavulanic acid	Clindamycin	Mode 5
Alonso-Ezpeleta et al. 2018 [32]	EN	67	8 (11.9)	Amoxicillin + clavulanic acid	Amoxicillin	Clindamycin	Mode 7 Mean 6.8 ± 1.2
Babar et al. 2022 [33]	GDP	216	188 (87.0)	Amoxicillin	Metronidazole	Clindamycin	Mode 5
Bolfoni et al. 2018 [34]	EN	615	7 (1.1)	Amoxicillin	Azithromycin	Clindamycin	Mode 7
Dias et al. 2022 [31]	GDP	193	54 (28.0)	Amoxicillin	Amoxicillin + clavulanic acid	Clindamycin	Mode 7
Dias et al. 2022 [31]	EN	109	10 (9.2)	Amoxicillin	Amoxicillin + clavulanic acid	Clindamycin	Mode 7
Domínguez-Domínguez et al. 2021 [4]	GDP	190	84 (44.0)	Amoxicillin ± clavulanic acid	Metronidazole	Clindamycin	Range 5–7 Mean 6.2 ± 1.4
Drobac et al. 2021 [35]	GDP	158	2 (1.3)	Amoxicillin	Clindamycin	Clindamycin	Mode 5
Iqbal et al. 2015 [36]	GDP	157	43 (27.3)	Amoxicillin + clavulanic acid	Amoxicillin	Clindamycin	Not specified
Maslamani & Sedeqi 2018 [37]	GDP	227	69 (30.4)	Amoxicillin	Clindamycin	Clindamycin	Range 5–7
Mengari et al. 2020 [38]	GDP	310	37 (11.9)	Amoxicillin	Amoxicillin + Metronidazole	Clindamycin	Range 5–7
Özmen & Şahin 2024 [39]	GDP	343	34 (10.0)	Amoxicillin	Amoxicillin + clavulanic acid	Clindamycin	Range 5–7
Yaqoob et al. 2024 [30]	GDP	409	239 (58.4)	Amoxicillin + clavulanic acid	Metronidazole	Clindamycin	Mode ≥5

AB: antibiotic; GDP: general dental practitioner. EN: endodontist. Treatment duration is indicated in different forms by different investigators; (a) mean ± SD, (b) mode, (c) range.

**Table 6:** Risk of bias assessment using the modified Newcastle–Ottawa Scale (NOS) for prevalence studies.

Study (Author, Year)	1	2	3	4	5	6	7	8	Total	Risk of Bias Level
Alattas & Alyami 2017 [29]	*	*		*	*	*	*	?	6	Moderate
Alonso-Ezpeleta et al. 2018 [32]	*	*		*	*	*	*	?	6	Moderate
Babar et al. 2022 [33]	?			?	*		*		2	High
Bolfoni et al. 2018 [34]	*	*	*		*	*	*	?	6	Moderate
Dias et al. 2022 [31]	?			?	*	*	*	?	3	High
Domínguez-Domínguez et al. 2021 [4]	?	?		*	*	*	*	*	5	Moderate
Drobac et al. 2021 [35]	?	?			*	*	*	*	4	High
Iqbal 2015 [36]	?	?		*	*		*		3	High
Maslamani & Sedeqi 2018 [37]	?	*	*	?	*	*	*	*	6	Moderate
Mengari et al. 2020 [38]	?	?			*	*	*	*	4	High
Özmen & Şahin 2024 [39]	*	*	*	*	*	*	*	*	8	Low
Yaqoob et al. 2024 [30]	?			?	*	?	*		2	High
Total	4	5	3	5	12	9	12	5	55	Moderate

1. Representativeness of the sample; 2. Sampling frame and selection method; 3. Sample size justification; 4. Non-response bias; 5. Case/phenomenon definition; 6. Measurement tool validity; 7. Consistency of data collection; 8. Statistical analysis. The asterisk (*) indicates that the study met the criterion; in doubtful cases, a question mark (?) is indicated.

**Table 7:** GRADE assessment of evidence quality.

GRADE Domain	Judgment	Reason for Downgrade	Comments
Risk of bias	Serious ↓	Most studies were cross-sectional surveys with methodological limitations; majority judged as moderate to high risk of bias; only one low-risk study.	Downgraded one level.
Inconsistency	Very serious ↓↓	Extreme heterogeneity (I^2^ = 98%, *p* < 0.0001); prevalence ranged from ~1% to 87%; heterogeneity unexplained by subgroup analyses.	Downgraded two levels.
Indirectness	Serious ↓	All data based on self-reported practices; diagnosis often not strictly defined; variability in healthcare contexts across countries impacts applicability.	Downgraded one level.
Imprecision	Serious ↓	Wide 95% CI (10.4–32.6%); several studies with small sample sizes or low event numbers.	Downgraded one level.
Publication bias	Serious ↓	Funnel plot suggested asymmetry; Egger’s test significant (*p* = 0.034); LFK index = +1.55 (minor asymmetry).	Downgraded one level.
Overall certainty of evidence	VERY LOW ↓↓↓↓	Cumulative downgrades across all domains.	The true prevalence may differ substantially from the pooled estimate.

## Data Availability

No new data were created or analyzed in this study. Data sharing is not applicable to this article.
